# Is combined peritoneal dialysis and hemodialysis redundant? A nationwide study from Taiwan

**DOI:** 10.1186/s12882-020-01989-1

**Published:** 2020-08-15

**Authors:** Mu-Chi Chung, Tung-Min Yu, Ming-Ju Wu, Ya-Wen Chuang, Chih-Hsin Muo, Cheng-Hsu Chen, Chao-Hsiang Chang, Jeng-Jer Shieh, Peir-Haur Hung, Jein-Wen Chen, Chi-Jung Chung

**Affiliations:** 1 Division of Nephrology, Department of Medicine, Taichung Veterans General Hospital, Taichung, Taiwan; 2 Program in Translational Medicine, National Chung Hsing University, Taichung, Taiwan; 3 Rong Hsing Research Center for Translational Medicine, National Chung Hsing University, Taichung, Taiwan; 4 Management Office for Health Data, China Medical University and Hospital, Taichung, Taiwan; 5 Department of Urology, China Medical University and Hospital, Taichung, Taiwan; 6 Department of Medicine, College of Medicine, China Medical University and Hospital, Taichung, Taiwan; 7 Institute of Biomedical Sciences, National Chung Hsing University, Taichung, Taiwan; 8 Department of Education and Research, Taichung Veterans General Hospital, Taichung, Taiwan; 9 Department of Internal Medicine, Ditmanson Medical Foundation Chiayi Christian Hospital, Chiayi, Taiwan; 10 Department of Applied Life Science and Health, Chia-Nan University of Pharmacy and Science, Tainan, Taiwan; 11 Department of Food and Beverage Management, Cheng Shiu University, Kaohsiung, Taiwan; 12 Center for Environmental Toxin and Emerging-Contaminant Research, Cheng Shiu University, Kaohsiung, Taiwan; 13 Super Micro Mass Research and Technology Center, Cheng Shiu University, Kaohsiung, Taiwan; 14 Department of Public Health, College of Public Health, China Medical University, No. 91 Hsueh-Shih Road, Taichung, 40402 Taiwan; 15 Department of Medical Research, China Medical University Hospital, Taichung, Taiwan

**Keywords:** Peritoneal dialysis, Hemodialysis, Combined therapy, Mortality, Admission, Cohort study

## Abstract

**Background:**

Combined peritoneal dialysis (PD) and hemodialysis (HD) therapy (combined therapy) has numerous clinical benefits and should be emphasized for PD patients encountering technique failure.

**Methods:**

This 12-year nationwide retrospective study was conducted to compare long-term outcomes (including admission and mortality risks) between combined therapy patients (combined group) and patients directly transferred from PD to HD (transfer group).

**Results:**

All 12,407 incidental PD patients from 2000 to 2010 were enrolled and followed up until the end of 2011. A total of 688 patients in the combined group and 688 patients in the transfer group were selected after 1:1 frequency matching based on age, sex, and PD duration. The overall admission and mortality risks of the two groups were comparable in a Cox proportional hazards model (adjusted hazard ratio [HR] = 1.06 [95% confidence interval (CI) = 0.95–1.19] and 1.02 [95% CI = 0.80–1.30]), respectively). Compared with the transfer group, combined group patients with recent peritonitis or frequent hemodialysis (four HD sessions per month) had significantly higher risk of admission while combined group patients without peritonitis had significantly lower risk. The number of incidents in the combined group increased over time. On average, patients stayed on combined therapy for 2 years.

**Conclusions:**

Combined therapy (two HD sessions per month) is not redundant but a rational and cost-effective treatment, particularly for patients without recent peritonitis. Dialysis staff should be familiar with the advantages and disadvantages of combined therapy and consider it an essential part of integrated dialysis care.

## Background

Peritoneal dialysis (PD) and hemodialysis (HD) are complementary rather than competitive dialysis therapies. PD has benefits as an initial modality due to its association with improved survival during the first 2–3 years [1], lower cost, and greater self-reported quality of life [2]. Accordingly, a policy of integrated dialysis care with PD first and then HD has been established [3]. Technique failure requires 10% of patients with PD per year to switch to HD [4] and the timely transfer of PD patients to HD is important for long-term survival [5]. However, as bridge therapy between full-time PD and full-time HD, the role of combined PD and HD therapy (combined therapy) is always overlooked.

Combined therapy was first proposed in 1996 by Kimura and Watanabe in Japan [6]. Due to their advantages, this therapy, generally comprising 5–6 days of PD and one HD session per week, overcomes the shortcomings of PD and HD and is more acceptable than direct transfer to HD. Several studies showed that combined therapy could increase dialysis adequacy, decrease fluid overload, improve peritoneal membrane function, and ultimately boost quality of life [6–9]. However, these single-center studies were limited by small patient numbers and focused on before versus after comparisons. One single-center study indicated an equivalent mortality risk for combined therapy, HD alone, and PD alone [10].

The Taiwanese National Health Insurance (TNHI) system provides services for HD and PD, including the use of icodextrin, Nutrineal, and automated PD. The TNHI system has also expanded coverage to combined therapy, but only if the symptoms of uremia and fluid overload cannot be ameliorated by maximizing the PD prescription. PD patients can decide whether to directly transfer to HD or to receive combined therapy.

To the best of our knowledge, our investigation is the first nationwide study of combined therapy. The present study aimed to explore the current practice of combined therapy and to clarify the long-term prognosis for mortality and admission risks if PD patients select it as bridge therapy.

## Methods

### Data sources

We used a longitudinal health insurance database of catastrophic illness patients (LHID-CIP) for this retrospective cohort study. The LHID-CIP was obtained from the TNHI program released by the Taiwanese National Health Insurance Administration, Ministry of Health and Welfare. Thirty disease categories were included in the registry of catastrophic illness patients in Taiwan, including malignancy, coagulation defects, end-stage renal disease (ESRD), and type I diabetes. Based on TNHI guidelines, a patient was defined as a catastrophic illness patient (CIP) by a specialist and by clinical reports such as pathological reports, blood tests, and kidney function tests. The LHID-CIP contained medical claims and patient information from 1997 to 2011. LHID-CIP diseases were defined according to the International Classification of Diseases, Ninth Revision, Clinical Modification (ICD-9-CM), which was established by the World Health Organization. To protect personal information, this study was approved by the Research Ethics Committee at China Medical University and Hospital (CMUH104-REC2–115-CR-4).

### Study participants

All 12,407 ESRD patients (ICD-9-CM code 585) who received PD from 2000 to 2010 were collected. Patients aged < 18 years were excluded, as well as those with a history of kidney transplantation, stayed on PD, and death or kidney transplantation on PD. The patients were divided into two groups: a combined therapy group (combined group) and a group comprising those directly transferred to HD (transfer group). Patients who were undergoing regular PD with at least two HD sessions per month at the outpatient department were included in the combined group. Patients who stopped PD and permanently transferred to HD treatment at the outpatient department were included in the transfer group. For added comparability, the transfer group was frequency matched with the combined group according to age, sex, and duration on PD (time from PD to HD initiation). The date of HD initiation was defined as the index date. The details are shown in Supplementary Figure 1.

### Outcomes and risk factors

The outcomes included mortality and incidence of admission. Admission included both hospitalization and emergency department visit. All participants were followed up from the index date until the date of mortality or admission. Those without outcome occurrence were followed up until the date of withdrawal from the TNHI program or the end of 2011. Risk factors considered in this study included age, sex, year of PD, duration on PD, Charlson comorbidity index, baseline comorbidity (including diabetes, hypertension, ischemic heart disease, chronic heart failure, cerebrovascular disease, peripheral arterial occlusive disease, and malignancy), medical treatment use (including automated PD and icodextrin use within 3 months before the index date), recent peritonitis (peritonitis diagnosis within 3 months before the index date), and type of vascular access for HD (including arteriovenous fistula/arteriovenous graft and tunneled catheter) before initiation of hemodialysis. Temporary double lumen catheter was used for hemodialysis by patients who did not have prepared vascular access.

### Reasons for admission

Reasons for admission were divided into peritonitis (ICD-9-CM codes 996.68, 567.9, and 567.2), infection due to vascular devices, implants, and grafts (ICD-9-CM code 996.62), infection other than peritonitis and vascular devices (ICD-9-CM codes 999.3, 599.0, and 486), other complications of PD (ICD-9-CM codes V562, 996.56, and 550.90), other complications of vascular devices (ICD-9-CM codes 996.1 and 996.73), and coronary artery disease (ICD-9-CM codes 414.01, 428.0, and 518.4).

### Statistical analysis

All statistical analyses were performed using SAS software version 9.4 (SAS Institute, Cary, NC), and the statistical significance level was set at *p* < 0.05 with a two-tailed test. Categorical variables are presented as number and percentage. Differences between the two groups were determined using a chi-square test or Fisher’s exact test when the cell number was less than five. Continuous variables are shown as the mean (standard deviation [SD]) and median (quartile 1 [Q1] and quartile 3 [Q3]), and differences between the two groups were determined by Student’s *t*-test and Wilcoxon rank-sum test. The mortality and incidence of admission were calculated according to the sum of the outcome number (death or admission) divided by the sum of person-years in both groups. A standard Cox proportional hazards model was used to assess the risk of death. Because the mortality differed between the two groups, we used a Cox proportional hazards model with competing risk of death to estimate the risk of admission. A multivariable Cox model was adjusted for age, sex, and variables with significant differences, as shown in Table 1. The stratified analysis was adjusted by age, duration of PD, Charlson comorbidity index, and recent peritonitis. Cumulative incidence of death and admission was plotted by Kaplan–Meier analysis, and a log-rank test was used to evaluate differences between the two groups. The distribution of the duration of PD and duration on hybrid therapy in the combined group was also presented. The propensity score matching method was used for sensitivity analysis in this study. The propensity score of the combined group and transfer group was calculated using stepwise logistic regression models which involved age, Charlson comorbidity index score, DM, use of icodextrin, type of vascular access for HD, and recent peritonitis.

**Table 1:** Demographic profiles of peritoneal patients in the combined and transfer groups

	Combined group						Transfer group (*N* = 688)		Combined group vs. Transfer group	Three groups
	HD^a^ ≤ 3 (*N* = 378)		HD^a^ = 4 (*N* = 310)		Total (*N* = 688)					
Variable	n	%	n	%	n	%	n	%	*p*-value	*p*-value
Age, years									1.00	0.67
< 65	316	83.6	251	81.0	567	82.4	567	82.4		
≥65	62	16.4	59	19.3	121	17.6	121	17.6		
Mean (SD)	49.1	(14.3)	51.6	(14.2)	50.2	(14.3)	50.2	(14.3)	0.91	0.08
Sex									1.00	0.04
Female	171	45.2	170	54.8	341	49.6	341	49.6		
Male	207	54.8	140	45.2	347	50.4	347	50.4		
Year at cohort entry									0.38	< 0.0001
Before 2004	114	30.2	15	4.84	129	18.8	142	20.6		
After 2004	264	69.8	295	95.2	559	81.3	546	79.4		
Duration of peritoneal dialysis, years									1.00	0.11
< 1 year	120	31.8	81	26.1	201	29.2	201	29.2		
1–2 years	82	21.7	68	21.9	150	21.8	150	21.8		
2–5 years	144	38.1	111	35.8	255	37.1	255	37.1		
> 5 years	32	8.47	50	16.1	82	11.9	82	11.9		
Mean (SD)	2.32	(1.96)	2.72	(2.17)	2.50	(2.06)	2.53	(2.17)	0.83	0.04
Charlson comorbidity index										
Median (Q1, Q3)	3	(2, 4)	3	(2, 4)	3	(2, 4)	3	(2, 4)	< 0.0001	< 0.0001
Mean (SD)	3.04	(1.76)	2.96	(1.78)	3.63	(1.77)	3.01	(1.77)	0.0002	0.0009
Comorbidity										
Diabetes	137	36.2	117	37.7	254	36.9	314	45.6	0.001	0.004
Hypertension	360	95.2	297	95.8	657	95.5	656	95.4	0.90	0.93
Ischemic heart disease	124	32.8	114	36.8	238	34.6	257	37.4	0.29	0.32
Chronic heart failure	88	23.3	74	23.9	162	23.6	194	28.2	0.049	0.14
Cerebrovascular disease	67	17.7	58	18.7	125	18.2	142	20.6	0.25	0.48
PAOD	27	7.14	27	8.71	54	7.85	53	7.70	0.92	0.74
Malignancy	24	6.35	23	7.42	47	6.83	48	6.98	0.92	0.85
Use of APD	72	19.1	85	27.4	157	22.8	173	25.2	0.31	0.02
Use of icodextrin	144	38.1	146	47.1	290	42.2	282	41.0	0.66	0.053
Recent peritonitis	95	25.1	118	38.1	213	31.0	344	50.0	< 0.0001	< 0.0001
Type of vascular access for HD									< 0.0001	< 0.0001
AVF/AVG	205	54.2	115	37.1	320	46.5	425	61.8		
Tunneled catheter	33	8.73	100	32.3	133	19.3	159	23.1		

*AVF* arteriovenous fistula, *AVG* arteriovenous graft, *APD* automated PD, *PAOD* peripheral artery occlusive disease

^a^*HD* hemodialysis sessions per month

## Results

From 2000 to 2010, 691 PD patients choose combined therapy as bridge therapy. The hemodialysis frequency per month was two (350 patients), three (28 patients), or four (310 patients). The numbers of incidents in the combined therapy patients were 4 in 2000, 81 in 2006, and 138 in 2010 (data not shown). The highest proportion of combined therapy was shown in the first year of PD (29.2%), and the proportion decreased in the year after PD (Supplementary Figure 2A). Calculation of the duration between the onset of combined therapy to the end, including pure HD, death, kidney transplantation, or the end of follow-up, revealed that about 42% of patients were maintained on combined therapy for less than 1 year and that patients maintained combined therapy for an average of 2.05 years (SD = 2.00) (Supplementary Figure 2B).

After frequency matching, 688 patients from the combined group and 688 patients from the transfer group were selected in this study. The mean age was 50.2 years (SD = 14.3) and there were slightly more males than females (50.4% vs. 49.6%) (Table 1). Compared with the transfer group, the combined group had a higher Charlson comorbidity index (*p* < 0.0001) but less commonly had diabetes (36.9% vs. 45.6%, *p* = 0.001), recent peritonitis (31.0% vs. 50.0%, *p* < 0.0001), and vascular access for HD (65.8% vs. 84.9%, *p* < 0.0001). Patients from combined group were further divided into two groups by hemodialysis frequency (HD ≤ 3 and HD = 4) (Table 1). Compared with HD ≤ 3 group, HD = 4 group had more females (54.8% vs. 45.2%), a higher usage of APD (27.4% vs. 19.1%), usage of icodextrin (47.1% vs. 38.1%), recent peritonitis (38.1% vs. 25.1%), use of tunneled catheter as vascular access for HD (32.3% vs. 8.73%).

**Fig. 1:**
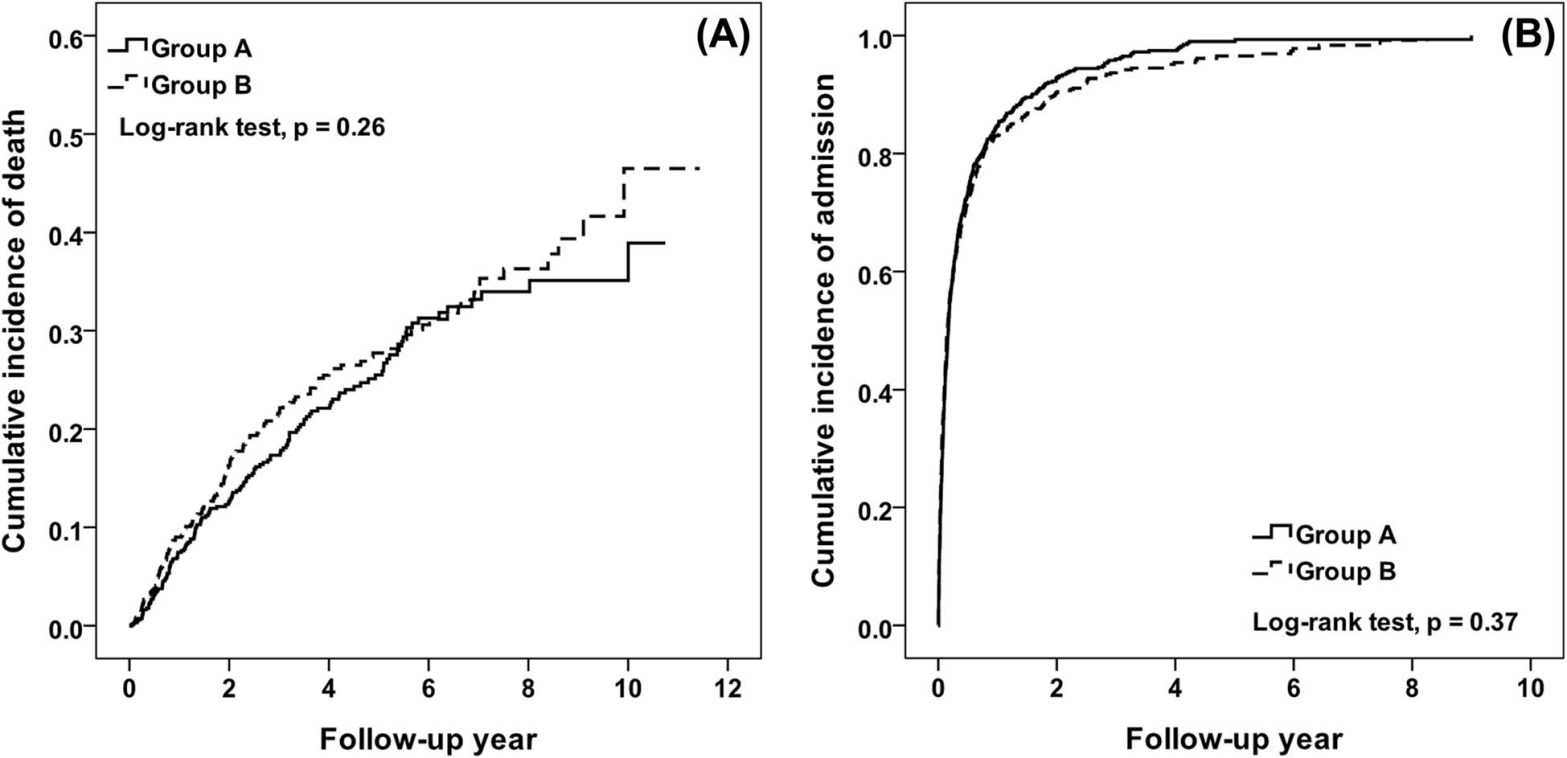
**a** Cumulative mortality risk and (**b**) admission risk of patients on combined therapy (combined group) and those who directly transferred to HD (transfer group)

During a 12-year follow-up, 145 and 155 patients died in the combined and transfer groups, giving mortality rates of 60.09 and 68.68 per 1000 person-years, respectively. Although the cumulative incidence of death was higher in the transfer group than in the combined group, it was not significantly different (Fig. 1a). Compared with the transfer group, the combined group had a similar risk of death (hazard ratio [HR] = 1.02, 95% confidence interval [CI] = 0.80–1.30) in a Cox proportional hazards model after adjustment for age, sex, diabetes, chronic heart failure, Charlson comorbidity index, recent peritonitis, and type of vascular access for HD (Table 2). When the combined group was stratified by frequency of HD, the risk of death was unchanged. All 646 and 628 patients exhibited an admission event, and the corresponding incidences were 1969.17 and 1656.15 per 1000 person-years in the combined and transfer groups, respectively. The cumulative incidence of admission was similar in the two groups according to Kaplan–Meier analysis (Fig. 1b). In a multivariable Cox proportional hazards model with competing risk of death, a similar risk of admission was found in the two groups (HR = 1.06, 95% CI = 0.95–1.19) (Table 2). Compared with the transfer group, combined group patients with four HD sessions per month had higher risk of admission (HR = 1.18, 95% CI = 1.03–1.35). Furthermore, we performed sensitivity analyses with propensity score matching method to compare the prognosis between combined group and transfer group (Supplementary Table 1) (Supplementary Table 2). In this analysis, outcome was similar to the primary analysis but there was no difference in risk of admission or mortality when the combined group was stratified by frequency of HD.

**Table 2:** Incidence rate ratios of admission and mortality risk according to HD frequency in the combined and transfer groups

Outcome	Event	Person-years	Rate	SHR (95% CI)	*p*-value
Admission					
Combined group					
Overall (*N* = 688)	646	328	1969.17	1.06 (0.95–1.19)	0.32
HD sessions = 2 (*N* = 350)	328	209	1570.58	0.96 (0.84–1.10)	0.56
HD sessions = 3 (*N* = 28)	26	13	2059.98	1.11 (0.74–1.66)	0.61
HD sessions = 4 (*N* = 310)	292	107	2739.33	1.18 (1.03–1.35)	0.02
Transfer group	628	379	1656.15	Ref.	
Outcome	Event	Person-years	Rate	HR (95% CI)	*p*-value
Mortality					
Combined group					
Overall (*N* = 688)	145	2413	60.09	1.02 (0.80–1.30)	0.88
HD sessions = 2 (*N* = 350)	79	1430	55.23	1.02 (0.77–1.37)	0.88
HD sessions = 3 (*N* = 28)	6	116	51.66	1.31 (0.57–2.99)	0.52
HD sessions = 4 (*N* = 310)	60	867	69.24	1.00 (0.73–1.36)	0.98
Transfer group	155	2257	68.68	Ref.	

Rate, per 1000 person-years; *SHR* subdistribution HR

Adjusted for age, sex, diabetes, chronic heart failure, Charlson comorbidity index, recent peritonitis, and type of vascular access for HD

**Fig. 2:**
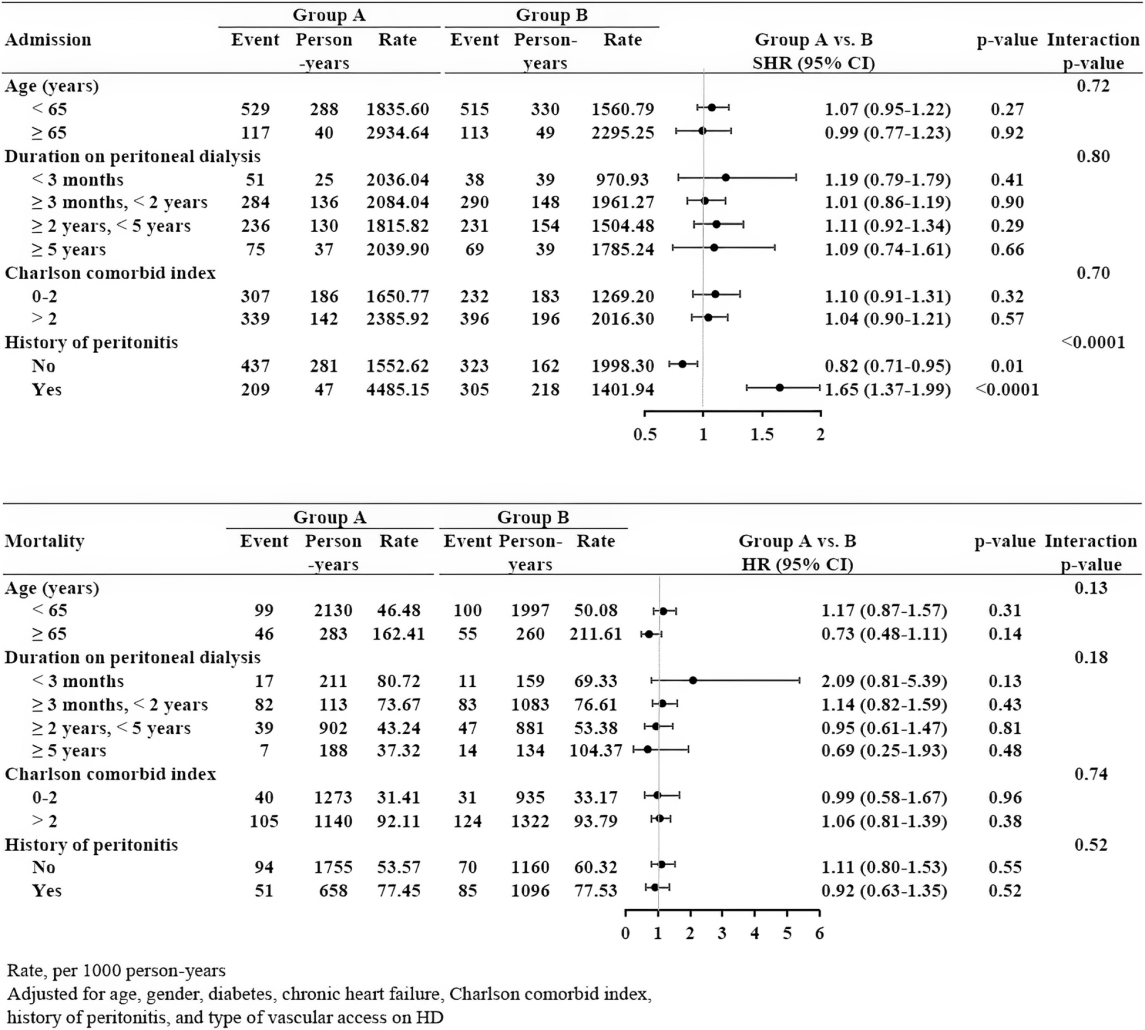
Incidence rate ratios of admission and mortality risk among the combined and transfer groups stratified by age, duration on peritoneal dialysis, Charlson comorbidity index, and recent peritonitis

Figure 2 presents the associations between outcomes and groups stratified by age, duration of PD, Charlson comorbidity index, and recent peritonitis. For admission events, the combined group exhibited a similar trend in the different subgroups compared with the transfer group, except in patients with recent peritonitis. In those with recent peritonitis, the combined group had significantly higher risk of admission than the transfer group (HR = 1.65, 95% CI = 1.37–1.99). However, in those without recent peritonitis, the combined group had significantly lower risk than the transfer group (HR = 0.82, 95% CI = 0.71–0.95) (interaction *p* < 0.0001). The different subgroups of the two groups had similar mortality rates (interaction *p* > 0.05).

Table 3 presents the incidence rate ratios of different reasons for admission between two groups. Compared with the transfer group, peritonitis was the main reason for admission and was significantly more common in the combined group after adjustment (HR = 2.06, 95% CI = 1.23–3.46) but admission for infection due to vascular devices, implants, and grafts was significantly lower in the combined group (HR = 0.46, 95% CI = 0.23–0.92).

**Table 3:** Incidence rate ratios of different reasons for admission between the combined and transfer groups

Outcome	Combined group			Transfer group			SHR (95% CI)	*p*-value
	Event	Person-years	Rate	Event	Person-years	Rate		
Peritonitis	49	328	149.36	25	379	65.93	2.06 (1.23–3.46)	0.006
Infection due to vascular devices, implants, and grafts	13	328	39.63	27	379	71.20	0.46 (0.23–0.92)	0.03
Infection other than peritonitis and vascular devices	31	328	94.50	26	379	68.57	1.22 (0.66–2.26)	0.53
Other complications of PD	56	328	170.70	49	379	129.22	0.88 (0.57–1.36)	0.56
Other complications of vascular devices	23	328	70.11	39	379	102.85	0.69 (0.40–1.21)	0.20
CAD	23	328	70.11	18	379	47.47	1.52 (0.79–2.95)	0.21

*CAD* coronary artery disease

Rate, per 1000 person-years. *SHR* subdistribution HR

Adjusted for age, sex, diabetes, chronic heart failure, Charlson comorbidity index, recent peritonitis, and type of vascular access for HD

## Discussion

This 12-year nationwide population-based follow-up study obtained several significant findings from patients receiving combined therapy. First, the overall mortality and admission risks of these patients were comparable to those who directly transferred to HD. Furthermore, in subgroup analysis, patients without recent peritonitis benefited from combined therapy due to lower admission risk. Finally, subsequent peritonitis was a major risk factor for admission. Combined therapy with two HD sessions per month was shown to be a good therapeutic option.

In the United States, conversion from PD to HD is frequent and occurs at a mean annual rate of 9.5% [4]. In a previous study, peritonitis was the most common cause of technique failure (average, 43%), followed by inadequate dialysis/ultrafiltration (average, 23%) [11]. Although we were unable to identify the actual cause of technique failure in our study, it can be deduced from our data on recent peritonitis and the PD regimen. Peritonitis (31 and 50% in the combined and transfer groups, respectively) and ultrafiltration failure were the causes of technique failure because icodextrin and automated PD were used by approximately 40 and 20% of patients in each group [12].

PD patients who experience technique failure could benefit both physically and psychologically from combined therapy. Physically, combined therapy improves dialysis adequacy and fluid overload. It also decreases body weight, blood pressure, serum creatinine, and even left ventricular mass index [13], as well as increases serum hemoglobin. Combined therapy may improve peritoneal membrane function by allowing a “PD holiday” entailing peritoneal rest [14]. Psychologically, combined therapy has a positive effect on health-related quality of life, including better physical function, fewer symptoms of kidney disease, and less of an impact of kidney disease on daily life [8]. It should also be emphasized that combined therapy may be more acceptable to patients because it permits a minimal lifestyle change and home-based continuous therapy.

When physicians are informing patients of technique failure and facing the transition, shared decision-making to determine individual preferences is important. It is necessary to explain the differences in the long-term outcomes of combined therapy and direct transfer to HD. Our study provides strong evidence of similar mortality and admission risks in the two groups after adjustment for potential confounders. Furthermore, we evaluated “life quality”, reflected by admission risk, because hospitalization is often followed by a decline in functional status that affects quality of life [15], and it appeared to be similar in the combined therapy and HD transfer groups.

For patients who transferred from PD to HD, the mortality risk peaked in the first year, which was attributed to infection (recurrent peritonitis/arteriovenous access infection) and cardiovascular events [16]. In our study, combined therapy was associated with a significantly higher peritonitis rate but lower vascular access infection than direct transfer to HD. It is worth emphasizing that peritonitis was the main negative factor for combined therapy. Not only was peritonitis associated with high admission risk, but also subsequent peritonitis was the main reason for admission in combined therapy patients. Recent peritonitis might increase the risk of repeat peritonitis [17] and aggravate ultrafiltration failure and membrane-related problems, which could partly explain the higher admission risk. We suggest that patients with recent peritonitis or high risk for peritonitis might be discouraged from combined therapy.

Because of the similar prognosis of combined therapy and transfer to HD, cost-effectiveness analysis is essential. For combined therapy, two HD sessions per month were covered by the TNHI system, but the other two sessions need to be paid by the patients themselves. Previous work reported PD and HD costs (US$/per year) in Taiwan of 17,723 and 21,367, respectively, including outpatient and inpatient expenses [18]. Each HD session costs $133 in Taiwan. Thus, combined therapy with two HD sessions per month costs $21,192 per year (26 HD sessions per year), which is still lower than the cost of pure HD; in contrast, combined therapy with four HD sessions per month is more expensive than pure HD. From insurance aspect, combined therapy with two HD sessions per month was an acceptable choice.

There is no doubt that four HD sessions per month were better for dialysis adequacy than two HD sessions per month, and the latter sometimes was regarded as rescue HD not combined therapy. Combined therapy with four HD sessions was unexpectedly associated with higher admission risk in our study, which might be not only related to complications with frequent hemodialysis, but also reflect the underlined difference between these two groups of patients. Compared to two HD sessions, combined group with four HD sessions had more usage of APD, icodextrin, recent peritonitis and more tunneled catheter as vascular access to HD. Combined therapy with two HD sessions per month was a feasible alternative from clinical and insurance perspective in Taiwan.

Given the advantages of combined therapy, the total number of patients in Japan on combined therapy increased from 600 in 2002 (5.5% of all PD patients) to 1900 patients in 2013 (20% of all PD patients) [10]. The use of combined therapy has increased in recent years in Taiwan but is still considerably lower than in Japan. Our dialysis staff should be familiar with the advantages and disadvantages of combined therapy and consider it an essential part of integrated dialysis care.

The strengths of this study include its nationwide scope and the fact that Taiwan is one of the few countries to adopt combined therapy. Furthermore, given the high coverage rate and continuity of the TNHI database, we were able to compare long-term outcomes between combined therapy and direct transfer to HD. Our findings are essential to determine the final piece in the puzzle of integrated dialysis care.

Some limitations of this study should also be considered. First, detailed laboratory results were not included in our database. We could not identify the albumin level, residual renal function, nutritional status, ultrafiltration rate, peritoneal function test, plasma β2-microglobulin level, hyperphosphatemia, and dialysis clearance (weekly Kt/V and weekly creatinine clearance), all of which might be associated with the outcome. Second, despite age, sex, and PD duration being matched between patients with combined therapy and those who transferred to HD, there were still residual biases between the groups, including indications and numerical data of changing the dialysis modality to combined or transfer therapy, fluid status, poor self-management of fluid balance, possibility of continuing PD therapy and the patients’ requests. Third, the combined therapy protocol, including the numbers of HD sessions per month and the length of PD holidays, might have varied among hospitals.

## Conclusions

Our study revealed that combined therapy is not a redundant but a rational and cost-effective therapy, particularly for patients without recent peritonitis. This study aimed to support evidence-based shared decision-making for PD patients facing the transition. Dialysis unit staff, including physicians and nurses, should be able to introduce combined therapy to patients as an ideal alternative choice.

## Supplementary information

**Additional file 1: Table S1.** Demographic profiles of peritoneal patients in the combined and transfer groups by propensity score matching method. **Table S2.** Incidence rate ratios of admission and mortality risk according to HD frequency in the combined and transfer groups by propensity score matching method.

**Additional file 2: Figure S1.** Flow diagram illustrating patient selection.

**Additional file 3: Figure S2.** (A) Distribution of the duration of PD at the start of combined therapy (years). (B) Distribution of the duration between the onset of combined therapy to the end, including transfer to HD, death, kidney transplantation, or the end of follow-up (years).

## Data Availability

The data that support the findings of this study are available from the Taiwan National Health Insurance Research Database (NHIRD) but restrictions apply to the availability of these data, which were used under license for the current study, and so are not publicly available. Any detail for data requests can be through the NHIRD (http://nhird.nhri.org.tw).

## Abbreviations

PD: Peritoneal dialysis
HD: Hemodialysis
ICD-9-CM: International Classification of Diseases Ninth Revision Clinical Modification
HR: Hazard ratio
