# Supplementary material for: Is combined peritoneal dialysis and hemodialysis redundant? A nationwide study from Taiwan
Source: BMC Nephrol. 2020 Aug 15;21:348. doi: 10.1186/s12882-020-01989-1 (PMC7429794; doi:10.1186/s12882-020-01989-1)
Supplement: Supplementary file 1 — Additional file 1: Table S1. Demographic profiles of peritoneal patients in the combined and transfer groups by propensity score matching method. Table S2. Incidence rate ratios of admission and mortality risk according to HD frequency in the combined and transfer groups by propensity score matching method. [file 12882_2020_1989_MOESM1_ESM.docx]

| Supplementary Table 1. Demographic profiles of peritoneal patients in the combined and transfer groups by propensity score matching method   \|  \| Combined group  (N=663) \| \| Transfer group  (N=663) \| \|  \| \| --- \| --- \| --- \| --- \| --- \| --- \| \| Variable \| n \| % \| n \| % \| p-value \| \| Age, years \|  \|  \|  \|  \| 0.78 \| \| <65 \| 542 \| 81.8 \| 538 \| 81.2 \|  \| \| ≥65 \| 121 \| 18.3 \| 125 \| 18.9 \|  \| \| Mean (SD) \| 504 \| (14.4) \| 50.9 \| (15.1) \| 0.61 \| \| Sex \|  \|  \|  \|  \| 0.07 \| \| Female \| 327 \| 49.3 \| 360 \| 54.3 \|  \| \| Male \| 336 \| 50.7 \| 303 \| 45.7 \|  \| \| Year at cohort entry \|  \|  \|  \|  \| 0.28 \| \| Before 2004 \| 126 \| 19.0 \| 111 \| 16.7 \|  \| \| After 2004 \| 537 \| 81.0 \| 552 \| 83.3 \|  \| \| Duration of peritoneal dialysis, years \|  \|  \|  \|  \| 0.21 \| \| <1 year \| 192 \| 29.0 \| 196 \| 29.6 \|  \| \| 1–2 years \| 145 \| 21.9 \| 145 \| 21.9 \|  \| \| 2–5 years \| 249 \| 37.6 \| 222 \| 33.5 \|  \| \| >5 years \| 77 \| 11.6 \| 100 \| 15.1 \|  \| \| Mean (SD) \| 2.49 \| (2.04) \| 2.57 \| (2.24) \| 0.49 \| \| Charlson comorbidity index \|  \|  \|  \|  \|  \| \| Median (Q1, Q3) \| 3 \| (2, 4) \| 3 \| (2, 4) \| 0.89 \| \| Mean (SD) \| 3.02 \| (1.79) \| 2.97 \| (1.67) \| 0.65 \| \| Comorbidity \|  \|  \|  \|  \|  \| \| Diabetes \| 251 \| 37.9 \| 240 \| 36.2 \| 0.53 \| \| Hypertension \| 636 \| 95.9 \| 625 \| 94.3 \| 0.16 \| \| Ischemic heart disease \| 235 \| 35.4 \| 246 \| 37.1 \| 0.53 \| \| Chronic heart failure \| 158 \| 23.8 \| 147 \| 22.2 \| 0.47 \| \| Cerebrovascular disease \| 122 \| 18.4 \| 131 \| 19.8 \| 0.53 \| \| PAOD \| 53 \| 7.99 \| 52 \| 7.84 \| 0.92 \| \| Malignancy \| 42 \| 6.33 \| 41 \| 6.18 \| 0.91 \| \| Use of APD \| 153 \| 23.1 \| 176 \| 26.6 \| 0.14 \| \| Use of icodextrin \| 280 \| 42.2 \| 277 \| 41.8 \| 0.87 \| \| Recent peritonitis \| 215 \| 32.4 \| 212 \| 32.0 \| 0.86 \| \| Type of vascular access for HD \|  \|  \|  \|  \| 0.99 \| \| AVF/AVG \| 322 \| 48.6 \| 325 \| 49.0 \|  \| \| Tunneled catheter \| 134 \| 20.2 \| 133 \| 20.1 \|  \|   AVF, arteriovenous fistula; AVG, arteriovenous graft; APD, automated PD; PAOD, peripheral artery occlusive disease.   \| Supplementary Table 2. Incidence rate ratios of admission and mortality risk according to HD frequency in the combined and transfer groups by propensity score matching method \| \| \| \| \| \| \| --- \| --- \| --- \| --- \| --- \| --- \| \| Outcome \| Event \| Person-years \| Rate \| SHR (95% CI) \| p-value \| \| Admission \|  \|  \|  \|  \|  \| \| Combined group \|  \|  \|  \|  \|  \| \| Overall (N=663) \| 624 \| 313 \| 1994.73 \| 0.98 (0.87-1.10) \| 0.32 \| \| HD sessions = 2 (N=332) \| 311 \| 196 \| 1590.47 \| 0.88 (0.76-1.01) \| 0.56 \| \| HD sessions = 3 (N=27) \| 26 \| 11 \| 2341.92 \| 1.14 (0.77-1.68) \| 0.61 \| \| HD sessions = 4 (N=304) \| 287 \| 106 \| 2702.90 \| 1.11 (0.97-1.28) \| 0.22 \| \| Transfer group \| 610 \| 319 \| 1914.64 \| Ref. \|  \| \| Outcome \| Event \| Person-years \| Rate \| HR (95% CI) \| p-value \| \| Mortality \|  \|  \|  \|  \|  \| \| Combined group \|  \|  \|  \|  \|  \| \| Overall (N=663) \| 143 \| 2314 \| 61.80 \| 0.95 (0.76-1.20) \| 0.88 \| \| HD sessions = 2 (N=332) \| 78 \| 1351 \| 57.75 \| 0.92 (0.70-1.21) \| 0.88 \| \| HD sessions = 3 (N=27) \| 6 \| 115 \| 52.34 \| 0.83 (0.36-1.94) \| 0.52 \| \| HD sessions = 4 (N=304) \| 59 \| 849 \| 69.53 \| 1.01 (0.75-1.37) \| 0.98 \| \| Transfer group \| 143 \| 2169 \| 65.93 \| Ref. \|  \| \|  \| \| \| \| \| \| |
| --- | --- | --- | --- | --- | --- | --- | --- | --- | --- | --- | --- | --- | --- | --- | --- | --- | --- | --- | --- | --- | --- | --- | --- | --- | --- | --- | --- | --- | --- | --- | --- | --- | --- | --- | --- | --- | --- | --- | --- | --- | --- | --- | --- | --- | --- | --- | --- | --- | --- | --- | --- | --- | --- | --- | --- | --- | --- | --- | --- | --- | --- | --- | --- | --- | --- | --- | --- | --- | --- | --- | --- | --- | --- | --- | --- | --- | --- | --- | --- | --- | --- | --- | --- | --- | --- | --- | --- | --- | --- | --- | --- | --- | --- | --- | --- | --- | --- | --- | --- | --- | --- | --- | --- | --- | --- | --- | --- | --- | --- | --- | --- | --- | --- | --- | --- | --- | --- | --- | --- | --- | --- | --- | --- | --- | --- | --- | --- | --- | --- | --- | --- | --- | --- | --- | --- | --- | --- | --- | --- | --- | --- | --- | --- | --- | --- | --- | --- | --- | --- | --- | --- | --- | --- | --- | --- | --- | --- | --- | --- | --- | --- | --- | --- | --- | --- | --- | --- | --- | --- | --- | --- | --- | --- | --- | --- | --- | --- | --- | --- | --- | --- | --- | --- | --- | --- | --- | --- | --- | --- | --- | --- | --- | --- | --- | --- | --- | --- | --- | --- | --- | --- | --- | --- | --- | --- | --- | --- | --- | --- | --- | --- | --- | --- | --- | --- | --- | --- | --- | --- | --- | --- | --- | --- | --- | --- | --- | --- | --- | --- | --- | --- | --- | --- | --- | --- | --- | --- | --- | --- | --- | --- | --- | --- | --- | --- | --- | --- | --- | --- | --- | --- | --- | --- | --- | --- | --- | --- | --- | --- | --- | --- | --- | --- | --- | --- | --- | --- | --- | --- | --- | --- | --- | --- | --- | --- | --- | --- | --- | --- | --- | --- | --- | --- | --- | --- | --- | --- | --- | --- | --- | --- | --- | --- | --- | --- | --- | --- | --- | --- | --- | --- | --- | --- | --- | --- | --- | --- | --- | --- | --- | --- | --- | --- | --- | --- | --- | --- | --- |
|  |

Rate, per 1000 person-years; SHR: subdistribution HR.

Adjusted for age, sex, diabetes, chronic heart failure, Charlson comorbidity index, recent peritonitis, and type of vascular access for HD
