# Supplementary figures and images for: Is combined peritoneal dialysis and hemodialysis redundant? A nationwide study from Taiwan
Source: BMC Nephrol. 2020 Aug 15;21:348. doi: 10.1186/s12882-020-01989-1 (PMC7429794; doi:10.1186/s12882-020-01989-1)

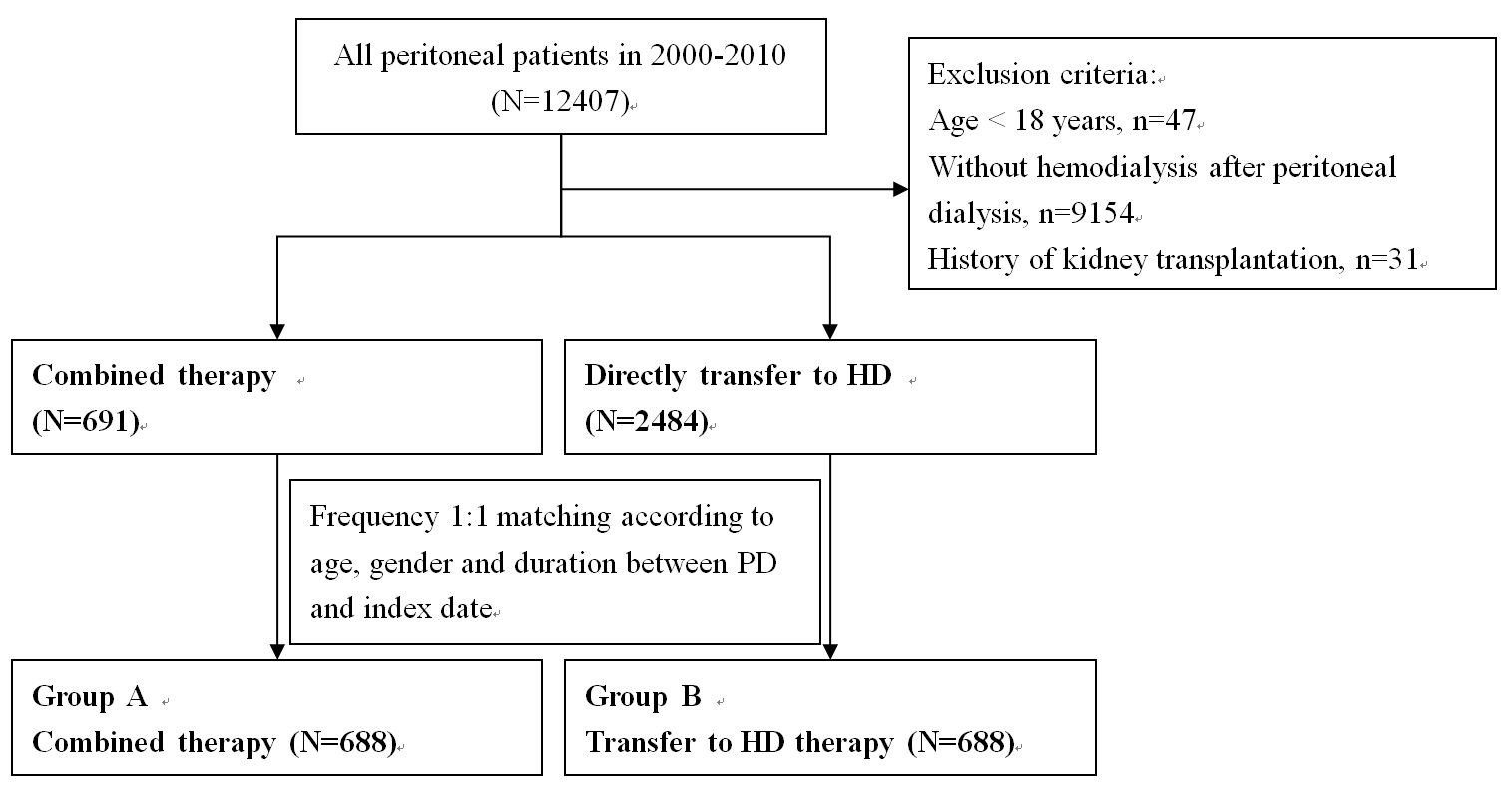

Supplement: Supplementary file 2 — Additional file 2: Figure S1. Flow diagram illustrating patient selection. [file 12882_2020_1989_MOESM2_ESM.tif]

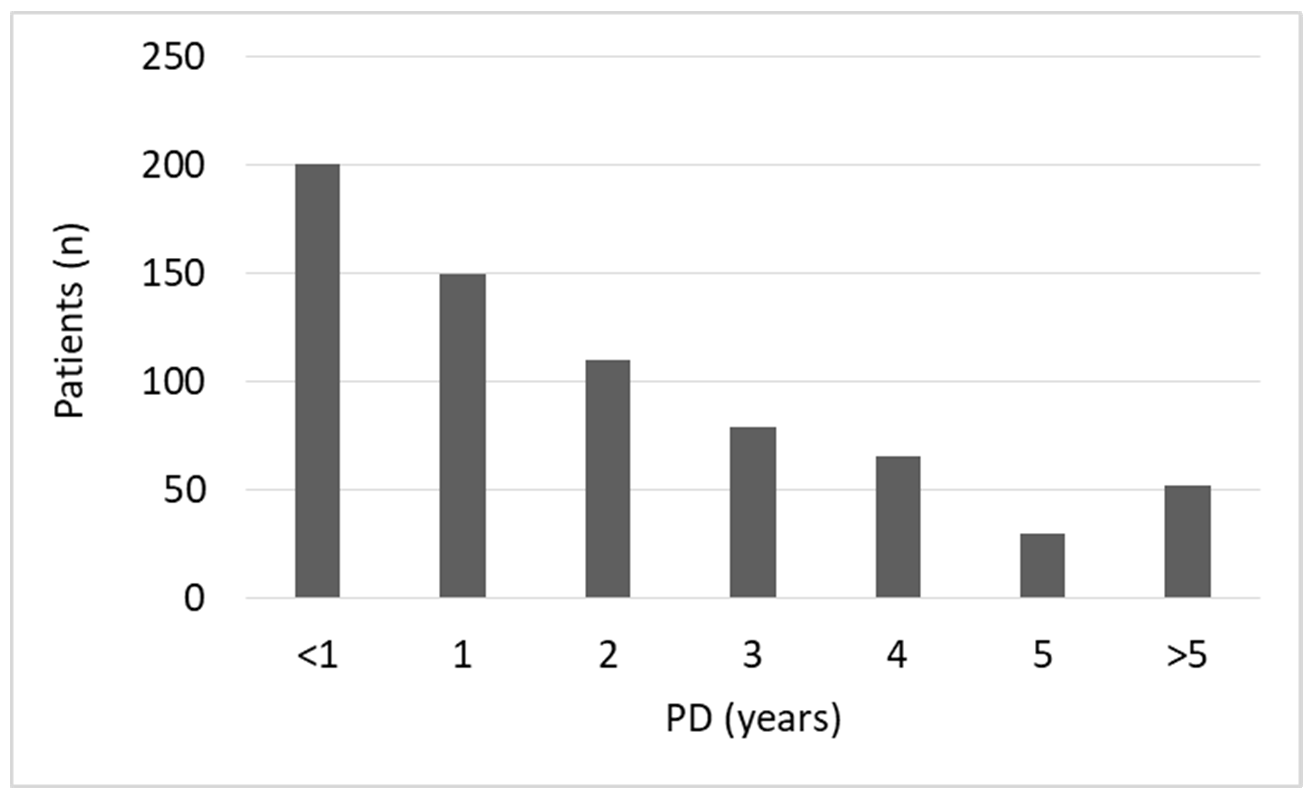

Supplement: Supplementary file 3 — Additional file 3: Figure S2. (A) Distribution of the duration of PD at the start of combined therapy (years). (B) Distribution of the duration between the onset of combined therapy to the end, including transfer to HD, death, kidney transplantation, or the end of follow-up (years). [file 12882_2020_1989_MOESM3_ESM.zip › suppl Fig 2 (A)R3.tif]

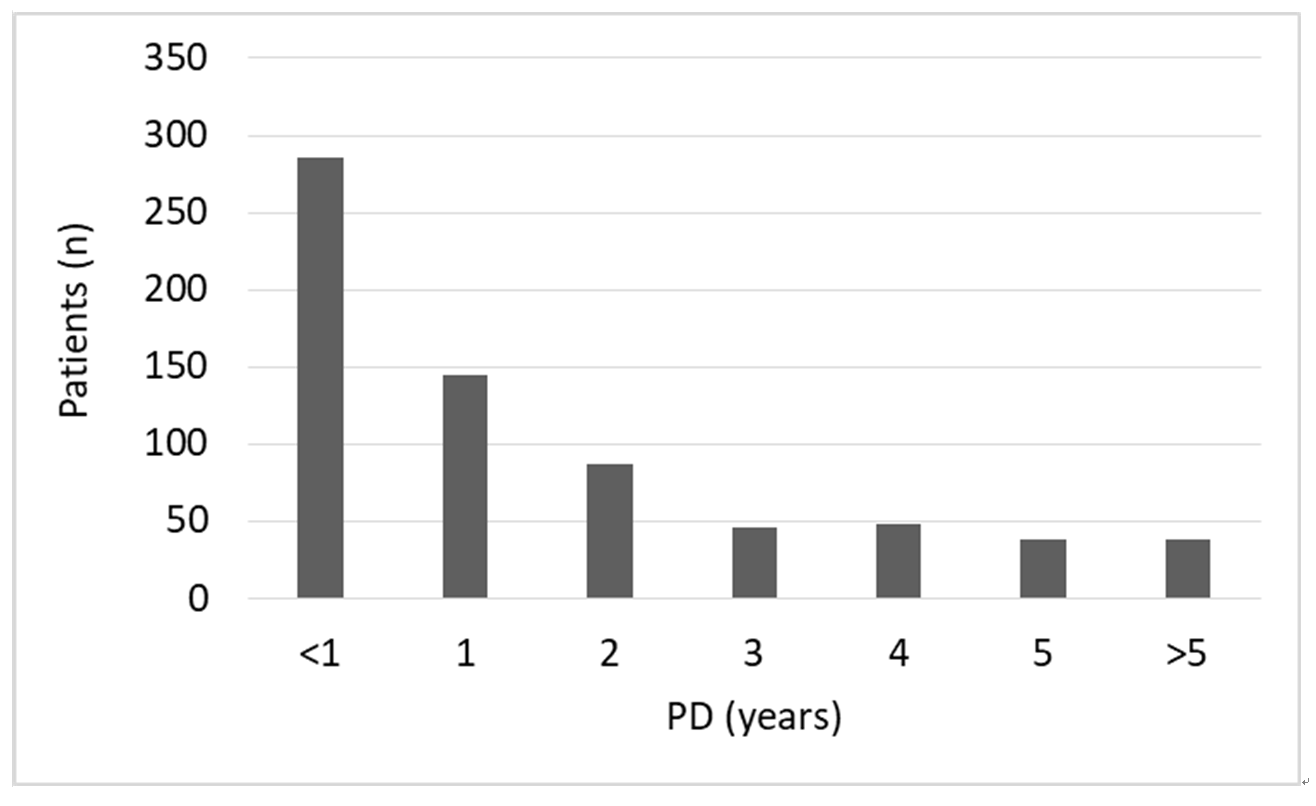

Supplement: Supplementary file 3 — Additional file 3: Figure S2. (A) Distribution of the duration of PD at the start of combined therapy (years). (B) Distribution of the duration between the onset of combined therapy to the end, including transfer to HD, death, kidney transplantation, or the end of follow-up (years). [file 12882_2020_1989_MOESM3_ESM.zip › suppl Fig 2 (B)R3.tif]
